# Our Boys

**Published:** 1860-10

**Authors:** 


### HALL'S JOURNAL OF HEALTH.

Our Legitimate Scope is almost boundless: for whatever begets pleasurable and harmless feelings, promotes Health; and whatever induces disagreeable sensations, engenders Disease.

WE AIM TO SHOW HOW DISEASE MAY BE AVOIDED, AND THAT IT IS BEST, WHEN SICKNESS COMES, TO TAKE NO MEDICINE WITHOUT CONSULTING A PHYSICIAN.

Vol. VII.] OCTOBER, 1860. [No. 10.

### OUR BOYS.

WHAT shall we make of them? What will become of them? These are practical questions, and made every day with serious solicitude by intelligent and thoughtful parents. The rich and the poor have a like ambition to put their sons in good places; they take more pains to select places which will honor their sons, than to make their sons capable of honoring places. The inquiry should be not for a place large enough for a son, but how to prepare a son to fill a place with profit to those who may call him to it, and with credit to himself.

An ancient and honored family-name in this city has been ineffaceably tarnished lately, by using family influence to get one of its members into a place of very high trust and responsibility; an office for which he was so utterly incompetent, that its accounts have fallen into inextricable confusion, while he himself, charged with a degrading crime, has been led in chains to a felon's cell, in a state of bodily health which melts the hardest heart with pity, while his venerable mother is made to weep tears of blood over the sad misfortunes of the child of her heart.

Inquire then what your child is fit for, rather than what will fit him; the Presidency of the Republic is fit for him, but he may not be fit for it; it may receive him, but he may not be able to fill it with ability and honor. That office is fit for any man, the greatest and the best, but your son might not be fit for it: to occupy it and fill it, to discharge its duties with fidelity. You must seek a place adapted to your son's capabilities, for you may not adapt his capabilities to a place. Seek a place for him which he will honor by elevating it, and making it the more influential; but do not seek to put him in a position which is to honor him. You are a rich man. It is neither safe nor respectable nor wise to bring any youth to manhood without a calling, without an occupation by which he could maintain himself in case he should lose his fortune. In looking around for such a calling, instead of making the inquiry what you would like him to become, seek rather to know what occupation is suited to his capacities—what calling his abilities can fill. You might well like him to become an eminent lawyer, but has he that plodding and that tenacity of purpose, which will enable him to investigate and compare and deduce with unerring accuracy for forty years, before he can be fairly able to commence practice? You might like for him to become a physician, but has he the self-denial to cut off the flesh from dead mens' bones, to live in the charnel-house for long years together; and then have the patience to wait for practice for other long years; and the self-sacrifice to go at every call, of prince or pauper, in the midnights of December, or the fierce suns of July, in rain or storm or sleet or snow? will he do this until forty years of age for a bare subsistence, before he can make patients come to him instead of he going to them?

Perhaps your heart burns to make him a minister, and in rapt imagination peering beyond the shores of time, you see him like some tall archangel leading along his vast battalions to the great white throne, saying, "Here am I, the instrumentality Thou hast made, of bringing these immortals here," and then loud peans come from serapic legions in glad reply, "Welcome, brother, Home!" No greater glory than this is there in earth or heaven for any created intelligence. But for such an office, it becomes a man that he have a range of learning beyond that of other men; has your son made the acquisition? He must have an abiding feeling that he is less than the least of all who love the Master, and must have the capacity to become all things to all men. Has he these humilities, and these versatilities? He must be silent when he is scorned; he must not return a stroke, nor answer to a taunt; when curses come he must bless; when sinned against he must forgive; has he the moral courage to meet these debasements, and yet above them all to stand and feel that he is second to no living man; that he is an ambassador from the court of the King of kings? Has he the breadth of intellect to compass all learnings? the humility of heart to feel abidingly before his Maker that he is but a worm, and yet the grandeur of soul in the light of the Lamb to feel, "I heir the universe by right of birth!"

Instead then of determining what you would like your son to be, seek to ascertain what he is capable of being; what he is certainly competent for. In short, seek not for your child the post he can get, but the post he can fill; for it is better to be an honor to the hod than a disgrace to the crown—better be an accomplished mechanic than a contemptible king!

